# Limnological Differences in a Two-Basin Lake Help to Explain the Occurrence of Anatoxin-a, Paralytic Shellfish Poisoning Toxins, and Microcystins

**DOI:** 10.3390/toxins12090559

**Published:** 2020-08-30

**Authors:** Zacharias J. Smith, Douglas E. Conroe, Kimberly L. Schulz, Gregory L. Boyer

**Affiliations:** 1 Ramboll, 333 W. Washington St., Syracuse, NY 13210, USA; 2 College of Environmental Science and Forestry, State University of New York, Syracuse, NY 13210, USA; kschulz@esf.edu; 3 Chautauqua Lake Association, Inc., Lakewood, NY 14750, USA; dougconroe481@gmail.com

**Keywords:** cyanotoxin co-occurrence, cyanobacteria, HABs, Chautauqua Lake

## Abstract

Chautauqua Lake, New York, is a two-basin lake with a deeper, cooler, and less nutrient-rich Northern Basin, and a warmer, shallower, nutrient-replete Southern Basin. The lake is populated by a complex mixture of cyanobacteria, with toxigenic strains that produce microcystins, anatoxins, and paralytic shellfish poisoning toxins (PSTs). Samples collected from 24 sites were analyzed for these three toxin classes over four years spanning 2014–2017. Concentrations of the three toxin groups varied widely both within and between years. During the study, the mean and median concentrations of microcystins, anatoxin-a, and PSTs were 91 and 4.0 μg/L, 0.62 and 0.33 μg/L, and 32 and 16 μg/L, respectively. Dihydro-anatoxin was only detected once in Chautauqua Lake, while homo-anatoxin was never detected. The Northern Basin had larger basin-wide higher biomass blooms with higher concentrations of toxins relative to the more eutrophied Southern Basin, however blooms in the North Basin were infrequent. Chlorophyll concentrations and toxins in the two basins were correlated with different sets of environmental and physical parameters, suggesting that implementing controls to reduce toxin loads may require applications focused on more than reductions in cyanobacterial bloom density (e.g., reduction of phosphorus inputs), and that lake limnological factors and morphology are important determinants in the selection of an appropriate management strategy. Chautauqua Lake is a drinking water source and is also heavily used for recreation. Drinking water from Chautauqua Lake is unlikely to be a significant source of exposure to cyanotoxins due to the location of the intakes in the deeper North Basin, where there were generally low concentrations of toxins in open water; however, toxin levels in many blooms exceeded the US Environmental Protection Agency’s recreational guidelines for exposure to cyanotoxins. Current cyanotoxin monitoring in Chautauqua Lake is focused on microcystins. However, the occurrence of blooms containing neurotoxic cyanotoxins in the absence of the microcystins indicates this restricted monitoring may not be sufficient when aiming to protect against exposure to cyanotoxins. The lake has a large number of tourist visitors; thus, special care should be taken to prevent recreational exposure within this group.

## 1. Introduction

Cyanobacteria produce several toxic compounds with a variety of chemical structures and biochemical activities [1,2,3,4]. Due to their widespread distribution in freshwater systems, the toxin classes of primary interest are the microcystins (MCs), the neurotoxic anatoxins (ATXs), including anatoxin-a, homo-anatoxin, dihydro-anatoxin, cylindrospermopsin, and related derivatives, and the paralytic shellfish poisoning toxins (PSTs). 

MCs are hepatotoxic peptides and are the primary focus of most cyanotoxin monitoring programs due to their common occurrence in freshwater environments. MCs have been reviewed in detail, including the global distribution of *Microcystis* [5] (a major toxin producer worldwide [6]), analytical methods for their detection [7], the toxicity and health effects of exposure [8], and several treatment and removal strategies for MCs [9]. There are over 250 congeners of MCs [10], with the most well-known congener being MC-LR, in which the two variable amino acids in the characteristic seven-membered peptide ring are leucine (L) and arginine (R). MCs are produced by several cyanobacterial genera, including *Dolichospermum* (basionym *Anabaena*), *Anabaenopsis*, *Aphanocapsa*, *Arthrospira*, *Hapalosiphon*, *Microcystis*, *Nostoc*, *Oscillatoria*, *Planktothrix*, *Snowella*, *Synechocystis*, and *Woronichinia* [8]. Several surveys for MCs have determined that congener profiles can vary spatially and temporally. A survey of 1161 lakes in 48 states in the United States that investigated seven MCs identified that the most common congeners in decreasing abundance were MC-LR, -YR, -RR, -LY, and -LA [11]. A smaller survey in 23 eutrophic midwestern lakes in the United State found the MC-LR, -RR, and -LA congeners were the most abundant MCs, with four congeners in detectable but less significant quantities [12]. A survey in New York found the most abundant congeners to be MC-LR, -RR, and -YR, with ten other detectable congeners having a low abundance [13]. Continuation of this survey in MCs in New York lakes has shown that congener profiles change between years, even within the same waterbody (unpublished).

Analytical methods for MCs include tandem mass spectrometry (Environmental Protection Agency (EPA) method 544) [14] and the ADDA (3-amino-9-methoxy-2,6,8-trimethyl-10-phenyl-4,6-decadienoic acid) ELISA (EPA method 546) [15], both of which are well-established methods. Other analytical methods include the protein-phosphatase inhibition assay [16], and HPLC with photodiode array detection and/or mass spectrometry [7]. The application of high-resolution mass spectrometry (HRMS is becoming an increasingly common tool for identifying new MC toxins [17].

The neurotoxic ATXs are known for their acute toxicity [18] due to their binding to nicotinic acetylcholine receptors [19]. The structure of anatoxin-a was elucidated in 1977 by Devlin et al. [20]. Subsequently, other anatoxin-a congeners have also been elucidated, including homo-anatoxin [21], dihydro-anatoxin [22], epoxy-anatoxin [23], epoxy-homoanatoxin [24], dihydro-homoanatoxin [25], and 4-hydroxyhomo-anatoxin [26]. ATXs are produced by members of the cyanobacterial genera *Dolichospermum*, *Cuspidothrix* (basionym *Aphanizomenon*), *Arthrospira*, *Cylindrospermum*, *Microcystis*, *Oscillatoria*, *Phormidium*, *Planktothrix*, and *Raphidiopsis* (basionym *Cylindrospermopsis*) [8]. While most analysis methods are based on tandem mass spectrometry (e.g., EPA method 545) [27], a commercial ELISA assay for anatoxin-a has been produced [28].

PSTs inhibit the sodium channel of higher organisms, including humans and marine mammals [29,30,31]. These toxins were originally discovered in marine dinoflagellates but are also produced by a range of cyanobacteria genera, including *Cuspidothrix, Dolichospermum*, *Microseira* (basionym *Lyngbya*), *Planktothrix*, *Raphidiopsis*, and *Scytonema*. PST-producing cyanobacteria are found in freshwater environments around the world, including in Australia [32,33], Brazil [34], United States [11,35,36,37,38,39,40], Canada [41], Germany [42], France [43], Portugal [44], Russia [45], and New Zealand [46]. Saxitoxin (STX), the parent compound in the PST group, is extremely toxic and shellfish are closely monitored for PSTs where the potential for human exposure might occur. Cases of human intoxication have been described in detail [47,48,49,50], where shellfish feeding on marine dinoflagellates and the bioaccumulation of PSTs can lead to lethal concentrations of toxins within shellfish tissues. There are more than 60 known congeners of STX [51,52,53]. Several of these STX congeners occur at higher molar concentrations than STX in freshwater and marine environments but are generally less toxic than STX, as measured using the mouse bioassay [36,54]. 

Multiple methods for PSTs have been utilized in recent decades, namely, the mouse bioassay (Association of Official Analytical Chemists (AOAC) method 959.08), the receptor binding assay (AOAC method 2011.27) [55], the pre-column (AOAC method 2005.06) [56] and post-column (AOAC method 2011.02) [57] AOAC certified methods for PSTs, and the internationally validated LC–MS/MS method [58], as well as other mass spectrometric methods reported in the literature [59]; multiple ELISAs for both neosaxitoxin and STX PST analogs have also been developed [60]. 

Cyanobacterial blooms containing MCs have led to the contamination of drinking water supplies in the United States, including Toledo, Ohio [8,61]; Skaneateles Lake, New York [62,63]; Owasco Lake, New York [64]; and Salem, Oregon [65], spurring attention at both state [66] and national levels, along with action plans for the management and reduction of cyanobacterial toxin contamination in water supplies. The United States EPA issued a 10 day guideline value for MCs in drinking water (0.3 μg/L for pre-school-aged children and 1.6 μg/L for school-aged children and adults) [67], and exceedances of these limits have caused health alerts for toxins in drinking water. While the EPA and several other regulatory bodies have reviewed toxicological information related with anatoxin-a, most have declined to issue guideline values for anatoxin-a and its derivatives due to missing toxicological information for both acute and long-term exposure [68,69,70], forcing regional authorities to create their own guidelines, resulting in a patchwork of regulations [71,72,73,74,75]. 

Anatoxins are produced by benthic cyanobacteria in New Zealand, where toxins produced by these organisms have contaminated drinking water supplies [25]. Anatoxins are widespread throughout New York lakes [13,76,77]; however, the presence of these toxins in finished water has not been evaluated. 

The toxicology of STX has been intensively evaluated since the mid-1960s due to the association of the compound and its derivatives with paralytic shellfish poisoning in coastal regions. The acute health risks from marine PSTs following the consumption of contaminated shellfish has been long-established [47,48,49,50]. Regulatory limits for total PSTs in shellfish are 80 µg STX eq./100 g of shellfish tissue, and these limits are widely adopted in the European Union, Australia, Canada, and the USA [78]. These guidelines were designed from studies evaluating the effects of acute exposure to toxins rather than chronic or sub-chronic exposure, which is an important route of exposure in freshwater systems. Additionally, most toxicological evaluations have focused on STX, while there is a dearth of toxicology data for the freshwater PST variants [36].

Few studies have evaluated cyanotoxin co-occurrence within the same waterbody. MCs and anatoxin-a were detected in rivers and streams in California and Pennsylvania in the United States [79,80,81], Argentina [82], Poland [83], Greece [84], Armenia [85], France [86], and in a wide survey of lakes across Europe [87]. Cyanobacteria also produce bioactive peptides other than MCs, which can co-occur with anatoxin-a [88,89] and MCs [90]. While synergistic effects from exposure to multiple cyanotoxins may increase the risk of exposure [91], only a limited number of assessments have evaluated the health effects of cyanotoxin mixtures [92].

**Figure 1:**
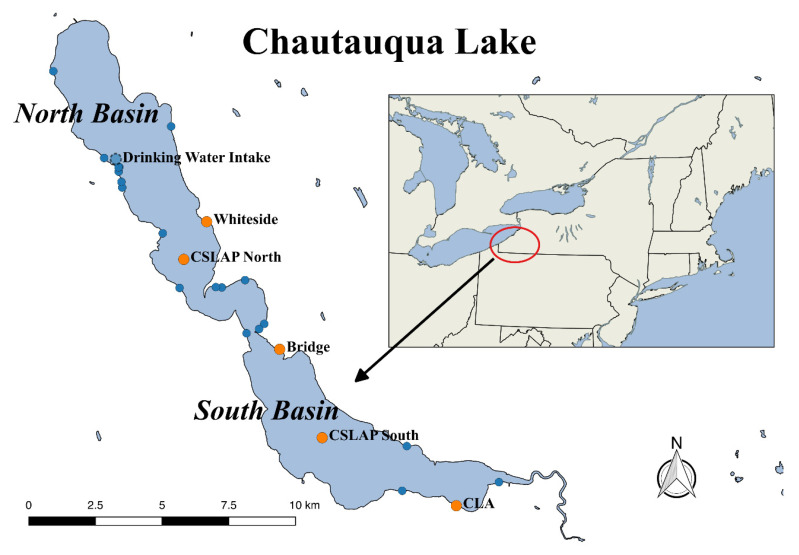
Map of Chautauqua Lake, New York (42°10′51.4″N, 79°25′50.5″W). The lake is divided into two basins with different trophic states. Two sites (Citizen’s Statewide Lake Assessment Program (CSLAP) North at 42°10′51.4″N, 79°25′50.5″ W, and CSLAP South at 42°07′23.8″N, 79°21′50.0″ W) were sampled eight times each year from 2014–2017 for basic water quality parameters. Three shoreline sites—Whiteside (42°11′38.5″N, 79°25′16.4″W), Bridge (42°09′08.4″N, 79°23′06.6″W), and CLA (42°06′09.5″N, 79°18′05.8″W)—were sampled weekly for toxins and cyanobacterial chlorophyll. Other shoreline sites, labeled in blue, were sampled when blooms were visually identified at the sites upon weekly inspection (Table S1).

Chautauqua Lake is a two-basin lake in western New York (42°10′51.4″N, 79° 25′50.5″W) (Figure 1), with algal blooms dominated by a range of cyanobacterial genera [93] that provide the potential for chronic and acute exposure to cyanotoxins. Potentially toxigenic cyanobacteria genera identified in the lake include *Cuspidothrix* (basionym *Aphanizomenon*), *Dolichospermum* (basionym *Anabaena*), *Microcystis*, and *Planktothrix*. The North Basin of the lake is the deeper and cooler of the two basins with an average depth of 9.1 m and a maximum depth of 23 m [93,94]. The North Basin stratifies, producing low oxygen conditions for large portions of the summer [95]. Comparatively, the South Basin has a maximum depth of 5.7 m and a mean depth of 4.7 m [93] and does not stratify for extended periods [95].

The complex mixture of cyanobacteria in the lake could lead to the co-occurrence of all three cyanotoxin classes: MCs, ATXs, and PSTs. Here, we evaluated the abundance of the three cyanotoxin classes between 2014–2017 and the frequency of toxin co-occurrence, as well as the number of blooms containing toxins with unsafe levels of cyanotoxins. We explored the correlation of cyanobacterial blooms and the three cyanotoxin classes to environmental and physical parameters in the whole lake, as well as within each sub-basin. We discuss the potential for exposure to these toxins and the associated risk through recreational contact or the consumption of contaminated drinking water, as well as the broader implications for human and animal health in lakes containing multiple cyanotoxins.

## 2. Results

### 2.1. Chautauqua Lake Water Quality and Algal Bloom Monitoring

Water quality measurements, total chlorophyll, and cyanobacterial chlorophyll measurements for the two basins of Chautauqua Lake are shown in Table 1. The South Basin was more eutrophic relative to the North Basin, where Secchi disk depths were on average 1 m shallower and the total phosphorus concentrations were 27.1 μg/L higher. The chlorophyll comparisons between the two basins were more complex. A summary of the highest quartile of blooms in the North and South Basins is shown in Table 2. While median cyanobacterial chlorophyll concentrations were lower in the North Basin relative to the South Basin, mean cyanobacterial chlorophyll concentrations were much higher in the North Basin. This difference was driven by several exceptionally large blooms in the North Basin, in which the maximum cyanobacterial chlorophyll concentration was ~10-fold higher than in the South Basin. Both mean total chlorophyll and cyanobacteria-specific chlorophyll concentrations were higher in the South Basin relative to the North Basin following natural log transformation. Blooms in Chautauqua Lake first formed in early July, with the blooms appearing in the South Basin approximately two weeks earlier than those in the North Basin [96]. A complex assemblage of algae was detected throughout the growing season in Chautauqua Lake (Table S2). Fewer than 5% of samples had only a single cyanobacterial taxon recorded, and complex mixtures of three or more cyanobacteria genera were common.

### 2.2. Occurrence of Cyanotoxins in Chautauqua Lake

The occurrence of MCs, ATXs, and PSTs in Chautauqua Lake is summarized in Table 3. MCs were widespread throughout Chautauqua Lake between 2014–2017. The MC congeners most frequently detected were MC-LR, -RR, and -YR, while trace congeners included MC-H_4_YR [97], -desmethyl-LR, -WR, -FR, and -LA (Figure S1). These latter congeners were detected at low concentrations and represented a small portion of the total MC concentration. The exception was H_4_YR in 2017, when concentrations of this variant were exceptionally high and accounted for ≈30% of the total MC concentrations. This congener was only detected during this year. 

Anatoxin-a was the predominant anatoxin congener detected in Chautauqua Lake; dihydro-anatoxin was detected in only 1/144 samples from 2016, while neither of dihydro-anatoxin nor homo-anatoxin were detected in 2017, nor were they detected in 2018 (data not shown). In contrast, PSTs were widespread in Chautauqua Lake. Most of the PST congeners detected were not readily identifiable based on existing standards. A single compound, possibly lyngbyatoxin 3 (LWTX 3) [36,98], was identified using high-resolution mass spectrometry [99] and may have been responsible for the high concentrations of PSTs in some samples. Samples not containing this LWTX-3-like compound had much lower concentrations of total toxins. Three of the eight samples that tested positive for PSTs using post-column oxidation (PCOX) were confirmed to contain PSTs using STX-ELISA, but concentrations determined using ELISA were lower than concentrations measured using PCOX (data not shown).

Four of these eight samples were also evaluated using a receptor-binding assay, where none contained PST bioactivity above the LOD of 2 μg STX eq./L.

Cylindrospermopsin and its derivatives, along with free β-methylaminoalanine (BMAA), were not detected among the years 2014–2017.

### 2.3. Basin-Wide Occurrence and Temporal Variation of Toxins

**Figure 2:**
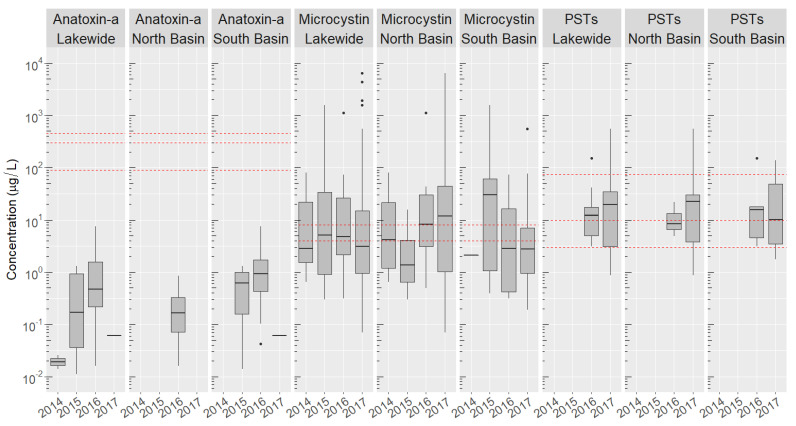
Box-and-whisker plots showing the log concentration ranges for microcystins, anatoxin-a, and total PSTs lakewide and in the North and South Basins of Chautauqua Lake over the years 2014–2017. PSTs were not analyzed in samples collected in 2014 and 2015. For years containing no box-and-whisker plot, no toxin was detected for that toxin in that year. Dashed red lines indicate different recreational thresholds set by several states and countries worldwide [72,73,74,75,102,103]. The upper and lower bounds of the boxes are the 25th and 75th percentiles, while the bar represents the mean. Whisker lengths are 1.5× the distance between the 25th and 75th percentiles, where any samples containing toxins outside this range are shown as individual points.

Cyanobacteria toxins were widespread in both basins of the Chautauqua Lake throughout multiple years (Figure 2, Figures S2 and S3). Blooms began producing toxins starting in July, with toxins remaining detectable in the water column through to the end of October. The three toxin classes were detected in both the North and South Basins, although not in all years or at all sites. 

While MCs were identified at higher frequencies in the eutrophic South Basin (20–40% of samples), the mean and median toxin concentrations were at times higher in the North Basin (Figure 2). The majority of samples contained between 1–100 μg/L of MCs, although some blooms contained much higher MC concentrations. Concentrations of toxins varied significantly from year to year in both basins. Almost no MC was detected in the South Basin in 2014, while conversely in 2015 MCs in the North Basin were found at much lower concentrations than in other years in both basins. More than half of the blooms exceeded the 4 μg/L recreational thresholds for MCs set by New York state [100], with far fewer exceeding the EPA threshold of 8 μg/L for MCs [101] (Figure 2).

Anatoxin-a was mostly associated with the Southern Basin, with 80% of all anatoxin-a detections occurring in this basin. The occurrence of anatoxin-a was highly variable between years, with 70% of all anatoxin-a detections occurring in 2016. This year was also the only year anatoxin-a was detected in the North Basin, although concentrations were 5–10-fold lower than those in the South Basin and anatoxin-a was detected at only one site. None of the three recreational thresholds [72,73,74] for anatoxin-a was exceeded.

PSTs were detected in both basins in 2016 and 2017. The concentrations of PSTs were slightly higher in the South Basin in 2016, but much higher in the North Basin in 2017. The number of PST-containing blooms was similar between the two basins over the two years. Most samples exceeded the 3 μg STX eq./L recreational guideline established by Ohio [72], and many exceeded the slightly higher 10 μg/L threshold set by Oregon [75], while few exceeded the least stringent 75 μg/L threshold set by Washington [102].

### 2.4. Site-Specific Occurrence of Toxins

**Figure 3:**
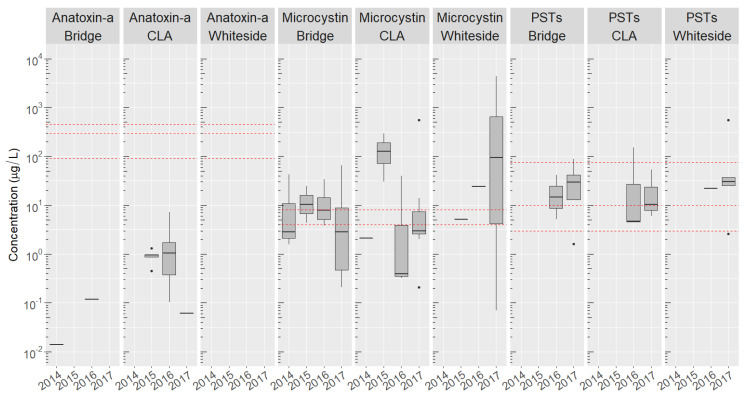
Box-and-whisker plots showing the log concentration ranges for microcystins, anatoxin-a, and total PSTs at the CLA, Bridge, and Whiteside sites over the years 2014–2017. PSTs were not analyzed in samples collected in 2014 and 2015. For years containing no box-and-whisker plot, the toxin was not detected for that toxin and year. Dashed red lines indicate different recreational thresholds set by several states and countries worldwide [72,73,74,75,102,103]. The upper and lower bounds of the boxes are the 25th and 75th percentiles, while the bar represents the mean. Whisker lengths are 1.5× the distance between the 25th and 75th percentiles, where any samples containing toxins outside this range are shown as points.

The detection frequency for MCs was highly variable between different sites of the lake. MCs were regularly detected at the Whiteside, Bridge, and CLA sites during the summer and fall of 2014–2017 (Figure 3). MCs were identified most commonly at the Bridge site, located between the two basins (Figure 1). The CLA site in the South Basin had slightly fewer MC detections compared to the Bridge, while the North Basin site, Whiteside, had the fewest MC detections and lowest mean toxin concentrations (Figure 3, Table S3).

An extreme bloom event occurred at the Whiteside site in 2017, where MC concentrations exceeded 100 μg/L in four of five samples collected over a five week period, with the maximum concentrations of toxins above 4000 μg/L. MCs were detected in all four years at the Bridge and CLA sites, with MCs appearing earlier in the season at these two sites compared to the Whiteside site. MC concentrations at the Bridge site were consistent between years, while MC concentrations at the CLA site were highly variable. Many blooms exceeded the New York state recreational guideline of 4 μg/L for MCs, although this heavily depended on the site and the year [100]. Far fewer blooms exceeded the 8 μg/L guideline at all locations [72].

While anatoxin-a was detected at the Bridge and CLA each of the four years, nearly half of all anatoxin-a-containing samples occurred at the CLA site in 2016, with a maximum measured concentration of 7.1 μg/L of anatoxin-a. No blooms exceeded the recreational guidelines for anatoxin-a established by California, Ohio, and Washington states [72,73,74].

PSTs were detected at all three sites more frequently than anatoxin-a, but less frequently than the MCs, with the prevalence and concentrations of PSTs similar in the years 2016 and 2017. Several blooms in late July or early August contained high concentrations of total PSTs, exceeding 100 μg/L in several blooms. The majority of blooms exceeded a 3 μg/L recreational guideline set by Ohio [72] and the 10 μg/L threshold set by Oregon [75]; however, few of the samples exceeded the 75 μg/L guidelines set by Washington [102].

### 2.5. Co-Occurrence of Multiple Cyanobacteria Toxins

**Figure 4:**
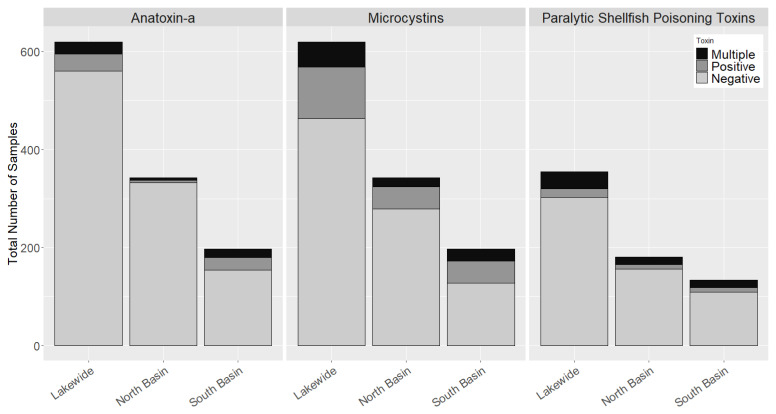
Bar plot representing the number of samples with two or more co-occurring cyanobacterial toxins in Chautauqua Lake between 2014–2017 across the whole lake, in the North Basin, and in the South Basin. Light grey sections represent samples that did not contain the primary toxin shown in figure header, the dark grey represents samples that contained the primary cyanotoxin, while black shows samples that contained the primary toxin in addition to one or both of the other two toxins.

As discussed above, MCs were detected with the greatest frequency lake-wide, and in both basins, they were followed by PSTs and anatoxin-a. However, PSTs were the most common toxin class to co-occur with another toxin class, followed by anatoxin-a and the MCs (Figure 4). This was the same in both basins. MCs were frequently found by themselves with only 15–30% of the MC-producing blooms also containing PSTs or anatoxin-a. In contrast, while PSTs and anatoxin-a were much less common than MCs, roughly half (30–60%) of the blooms containing these toxins also contained other toxins.

### 2.6. Correlation of Cyanobacterial Chlorophyll to Environmental Variables

Eleven environmental and physical parameters were evaluated for their correlation with cyanobacterial chlorophyll (Table 4). At a lakewide scale, the 11 terms were simplified to an ordinary least squares (OLS) model containing six parameters: photosynthetically active radiation (PAR), average wind speed (AWS), pH, total phosphorus (TP), conductivity, and total nitrogen (TN) (Figure S4A). Penalized regression models containing these six terms were developed and are shown in Figure S4B. Each of the selective models from the least selective ridge regression (α = 0) to the most selective least absolute shrinkage and selection operator (LASSO) (α = 1) converged on selecting pH, TP, and TN as the best predictors for cyanobacterial chlorophyll. The correlation of these parameters with cyanobacterial chlorophyll was primarily driven by the blooms that occurred in the North Basin, where the OLS model for the North Basin contained three of the five terms (AWS, pH, and TP) that were also selected in the lakewide OLS model.

The North and South Basins had different environmental parameters correlated with cyanobacterial chlorophyll concentrations. OLS models for cyanobacteria chlorophyll identified the AWS, pH, TP, TN, rainfall, and Secchi disk depth as predictors in the North Basin (Figure S4C), while in the South Basin, only TP was selected as a predictor (Figure S4E). OLS bootstrapped models were not selective, and many terms in the OLS models were not selected in penalized regression models. As selectivity in the OLS models was low, the identification of TP in the South Basin model did not indicate a strong correlation of TP with cyanobacterial chlorophyll. Penalized models in the North Basin (Figure S4D) selected three terms, namely, pH, TP, and Secchi disk depth, while a less stringent model also included rainfall. No penalized models were evaluated for cyanobacterial chlorophyll in the South Basin as only one term, namely, total phosphorus, was selected in the OLS model (Figure S4E).

### 2.7. Comparison between Environmental Parameters for Cyanobacterial Chlorophyll and the Three Cyanotoxins

The predictors for the three cyanotoxins were different from those for cyanobacterial chlorophyll (Table 4). MCs had only two parameters selected by the OLS model (Figure S5A) for the entire lake: conductivity and water temperature. Neither of these terms was selected in the penalized models. Similar to the cyanobacterial chlorophyll models in the North and South Basins, MCs in the two basins were correlated to different environmental parameters.

In the North Basin, the OLS model for MCs selected cyanobacterial chlorophyll, TP, conductivity, water temperature, rainfall, and Secchi disk depth as predictors (Figure S5B). The penalized MC models selected cyanobacterial chlorophyll, TP, water temperature, and rainfall when α = 0 (Figure S5C), where the predictors of cyanobacterial chlorophyll in the North Basin were pH, TP, Secchi disk depth, and rainfall.

In the South Basin, cyanobacterial chlorophyll, PAR, average wind direction (AWD), pH, and TP were selected by the OLS model for MCs (Figure S5D), with cyanobacterial chlorophyll, PAR, and AWD all retained at α = 0 (Figure S5E). Unlike the North Basin, the penalized models up to α = 0.5 still selected cyanobacterial chlorophyll as a predictor in the South Basin.

PSTs had four terms selected for the OLS model of the entire lake: cyanobacterial chlorophyll, AWS, pH, and conductivity (Figure S6A). Three of these terms—cyanobacterial chlorophyll, AWS, and conductivity—were retained in the α = 0 penalized regression model, but none of these terms were retained by the other more selective models (α > 0) (Figure S6B). For PSTs in the North Basin, the OLS model selected PAR, AWS, conductivity, water temperature, rainfall, and Secchi disk depth (Figure S7A), while the penalized models retained AWS, rainfall, and Secchi disk depth at α = 0 (Figure S7B). In the South Basin, the OLS PST model selected cyanobacterial chlorophyll, PAR, AWS, conductivity, water temperature, TN, rainfall, and Secchi disk depth (Figure S7C), with the α = 0 penalized model retaining cyanobacterial chlorophyll, AWS, rainfall, and Secchi disk depth (Figure S7D). 

In contrast to the MCs and PSTs, with the anatoxin-a OLS model for the entire lake selecting cyanobacterial chlorophyll, PAR, AWS, water temperature, TN, and rainfall (Figure S6C), several of these terms were retained in the most heavily penalized model (Figure S6D). Cyanobacteria chlorophyll and AWS were both retained in the α = 1 model, while PAR was also included at α ≤ 0.75, and all six terms selected in the OLS model were retained at α = 0. The same high level of correlation was not found in the models of the South Basin alone, where the OLS model for anatoxin-a selected cyanobacterial chlorophyll, PAR, AWS, pH, water temperature, and Secchi disk depth (Figure S7E), and only the α = 0 penalized model retained cyanobacterial chlorophyll, AWS, water temperature, and Secchi disk depth as predictors (Figure S7F).

## 3. Discussion

The two basins of Chautauqua Lake were found to be morphologically, chemically, and biologically different, which helped to explain the distinctly different blooms between basins. The South Basin was the more eutrophic of the two basins over the four years of analysis, where the concentrations of cyanobacterial chlorophyll were greater (Table 1), in part due to higher levels of total phosphorus and elevated surface water temperatures. These blooms were relatively consistent, appearing regularly and at similar densities between years. Unlike the South Basin, the North Basin was much more variable with infrequent but exceptionally large and highly concentrated blooms, which were not observed in the South Basin. These blooms were inconsistent, occurring only once over the measurement period of this study, with other recent basin-wide bloom events occurring twice in 2012 and 2013 [104].

Lake morphometry is highly influential in the formation and size of cyanobacterial blooms [105], where the differences in basin structures of Chautauqua Lake may have given rise to some of the differences observed in the blooms. Blooms in the deeper North Basin responded more to nutrient inputs, where rainfall and phosphorus concentrations were both found to be correlated with cyanobacterial chlorophyll. In contrast, there were no predictors of cyanobacterial chlorophyll in the shallow, nutrient-rich South Basin. Water temperature, while not found to be correlated to bloom size, was related to the onset of blooms, with blooms appearing several weeks earlier in the season in the warmer South Basin [106,107].

The environmental factors correlated to cyanobacterial chlorophyll were different between the North and South Basins, where nutrient terms were better correlated with cyanobacterial chlorophyll in the North Basin. Extrapolating beyond Chautauqua Lake, some lakes most closely resemble the North Basin, where there are episodic but large and highly toxic cyanobacteria blooms. Other shallow eutrophic water bodies can be better compared to the South Basin, where cyanobacterial blooms are a regular occurrence, but where there is less variability in the bloom density. Nutrient management plans are likely to be most effective in lakes most closely resembling the North Basin. While nutrient reduction plans are often proposed to reduce populations of cyanobacteria in highly productive lakes [108], in some of these lakes, nutrients may not be the primary limiter of growth. Alternative management approaches, such as the construction of new macrophyte communities, where macrophytes compete directly with cyanobacteria for light in addition to nutrients, may be more effective [109]. Importantly, “one-size fits all” approaches are unlikely to be effective at reducing cyanobacterial blooms.

Control strategies can assist in reducing the abundance of cyanobacteria [110], but may not necessarily reduce the concentrations of toxins and/or their toxicity. While cyanobacteria need to be present for there to be cyanotoxins, environmental and physical variables impact the presence of toxins independent of the density of a cyanobacterial bloom. While cyanobacteria bloom mitigation strategies may be effective at reducing bloom densities, toxin concentrations may not decrease proportionally with chlorophyll. MCs were correlated to nutrient parameters and water temperature in the North Basin, while in the nutrient-rich South Basin, neither of the three toxins were correlated to endogenous predictors, including nutrients. Instead, the three toxins were correlated with external physical parameters, including PAR, AWS, AWD, and water temperature (Table 4). The importance of these external factors may reduce the effectiveness of some mitigation strategies in reducing the concentrations of cyanobacterial toxins.

Between 2014–2017, toxin concentrations between basins and sites were highly variable, with the variability in toxin concentrations between years being greater than the variability within years. There was no trend between the concentration of toxins and the year. Although the variability was less in the South Basin compared to the North Basin, it remained high, where highly variable toxin concentrations have been observed in other shallow eutrophic lakes [111]. A high level of natural variability increases the difficulty of assessing the impact of an intervention on reducing cyanobacterial blooms, while also increasing the difficulty of assessing the impacts of the blooms on human and ecological health. Further study of toxins at Chautauqua Lake will continue to require intense sampling, similar to what was described in the study here, to account for variability in toxin content.

Sampling for the protection of human health in Chautauqua lake requires that samples be collected from all sites where the risks to recreational users are the highest. Toxin concentrations could be below recreational thresholds at one site and greatly exceed the threshold at other sites, even those nearby. While toxins were sometimes detected in open water, the highest concentrations of toxins were at shoreline sites, where physical parameters such as wind speed and direction, may have led to the accumulation of bloom material. At many times, the concentrations of toxins on the shorelines greatly exceeded the 4 µg/L recreational guideline for MCs in New York [100] and the 3 µg STX eq./L recreational guideline for PSTs in Ohio [72].

Exposure to cyanobacterial toxins through drinking water in the Chautauqua Lake region was less likely than recreational exposure. The drinking water intakes are located in the North Basin, where cyanobacteria blooms were less common, while the locations of the intakes were ~100 m offshore, limiting the potential for contamination from the densest blooms. While there were lake-wide blooms in the North Basin that would presumably lead to MCs and/or PSTs entering the water treatment plant [96], extreme blooms were infrequent such that exceedances would be rare, and that toxins would likely not remain past the 10 day criteria for MCs. In the case that toxins did enter the plant, many standard drinking water treatments are effective for MCs [9]. There are treatment strategies for ATXs and PSTs; however, many remain untested [112]. 

While symptoms of acute exposure to cyanotoxins have been described [19,113], only MCs have been evaluated and were found to be strongly linked to long term chronic health impacts [114,115]. The toxicology and neurological effects from chronic exposure to either ATXs or PSTs have not been evaluated. Whether periodic exposure to these toxins in drinking water is a cause for concern is unclear. Furthermore, the toxicological effects of cyanotoxins have been assessed individually, but not in combination. Synergistic or antagonistic effects of multi-cyanotoxin exposure have not been significantly evaluated [92]. Synergistic effects of these toxins could lead to underestimations of toxicity, as MCs regularly co-occurred with PSTs and anatoxin-a (Figure 4). Currently, most cyanobacteria toxin monitoring programs only incorporate MCs. MC monitoring alone may underestimate health risks from exposure to cyanobacteria toxins, as the presence of MCs did not capture many cases where only other cyanobacterial toxins were present.

The co-occurrence of multiple cyanobacterial toxins has been historically uncommon in the literature relative to the studies describing individual cyanobacterial toxins. In Chautauqua Lake, each of the three cyanobacterial toxin classes co-occurred with one another and at different rates depending on the location (Figure 4). While risk assessments are often made using one cyanobacterial toxin, usually MC-LR, the potential for combinatorial effects from exposure to multiple cyanobacterial toxins should not be discounted. Additionally, PSTs and anatoxin-a were detected without co-occurring MCs; therefore, estimates of exposure risk based only on MCs underestimate the danger to human and animal health in Chautauqua Lake. The quantification of these neurotoxins will be important in future monitoring efforts, as MCs alone do not adequately evaluate the presence of multiple cyanobacterial toxins. 

While *Microcystis* is a well-known producer of MCs and *Microcystis* were likely producing MCs in Chautauqua Lake [5], it is unclear whether other cyanobacterial genera were producing MCs concurrently throughout the study period. Furthermore, there are many known ATX and PST producers [8] that regularly co-occurred in blooms within Chautauqua Lake. Because complex mixtures of cyanobacteria were so common, it is challenging to identify which of the genera may have been producing the observed toxins. Furthermore, high concentrations of toxins may be produced by minor genera found within a bloom, as environmental and biochemical factors have been well established as important influences on the cellular toxin quota [40,116,117]. With the information collected for this study and without an additional genomic investigation [118], we could not evaluate which cyanobacteria were producing the neurotoxins in Chautauqua Lake. Complicating the issue, there is a limited amount of genetic information related to ATX production, making it challenging to perform genomic analysis.

The potential for exposure to cyanobacterial toxins cannot be estimated through the visual identification of specific genera. While many cyanobacteria produce toxins, the majority of blooms contained no detectable cyanotoxins when toxigenic taxa were identified, even in the many cases where there were high concentrations (>100 µg/L) of cyanobacterial chlorophyll. Risk assessments incorporating cyanobacterial genera and/or chlorophyll metrics significantly overestimate the risk of cyanotoxin exposure, which may cause detrimental impacts to tourism, which is critical to the regional economy of Chautauqua Lake.

The health risks posed by the neurotoxins are difficult to determine. For both the PSTs and anatoxin-a, guideline values for drinking water and recreational contact are highly variable. Anatoxin-a concentrations were generally low, but anatoxin-a was found to be unstable in natural environmental conditions [77,119,120]. Therefore, any ATX concentrations measured in the laboratory may underestimate the true concentrations of anatoxin-a in Chautauqua Lake itself. PSTs were commonly found in Chautauqua Lake. However, analysis of these toxins using a receptor binding assay found that there were no PSTs above the detection limit of the assay [121], suggesting that the PST congeners in the lake may be relatively non-toxic. A better understanding of the toxin profiles in freshwater systems, along with the potential for transformation [51,122] of freshwater PSTs to more toxic congeners, is needed to evaluate the potential risk from these toxins.

Chautauqua Lake contained a complex mixture of cyanobacterial toxins. The presence and concentrations of these toxins depended on the basin and site. The extreme heterogeneity of both cyanobacterial blooms and their associated toxins within and between years is problematic for protecting lake users from exposure. All sites where exposure is of concern need to be monitored, as concentrations of toxin frequently exceeded recreational guidelines. Monitoring should also include toxins other than the MCs, as the neurotoxic PSTs and anatoxin-a were also found within Chautauqua Lake. While some information about the toxicity of these cyanotoxins has been described, there is still significant uncertainty about the chronic toxicity of both neurotoxins, including the parent compounds and their derivatives. These neurotoxins may pose a significant risk to human and environmental health in other freshwater systems.

## 4. Materials and Methods

### 4.1. Sample Collection and Chlorophyll Analysis

Samples were collected biweekly from the North and South CSLAP sites (Figure 1) eight times over the summer and early fall each year between 2014–2017 using the methods described by the New York Department of Environmental Conservation [123]. Several water quality parameters, including TN, TP, water temperature, ammonia, nitrate plus nitrite, pH, Secchi disk depth, and conductivity, as well as cyanobacterial chlorophyll and toxin abundance, were collected over four years totaling 32 samples. Water quality measurement data were retrieved from the New York State Federation of Lake Association summary reports [124]. For mid-lake stations (CSLAP North at 42°10′51.4″N, 79°25′50.5″W and CSLAP South at 42°07′23.8″N, 79°21′50.0″W), particulate toxins were measured using 200 mL lake water samples filtered onto glass fiber filters (934-AH) and kept on ice during transport to the lab [123]. 

In addition to these mid-lake sites, composite samples for particulate and dissolved toxin analysis consisting of 250 mL of lake water were collected weekly from just below the surface of the water from the Whiteside (42°11′38.5″N, 79°25′16.4″W), Bridge (42°09′08.4″N, 79°23′06.6″W), and CLA (42°06′09.5″N, 79°18′05.8″W) sites beginning in May-June and ending October-November in all years. Samples were collected from locations marked in blue (Figure 1) one to five times per year using the same protocol, depending on whether a bloom was visually observed at each site during a weekly inspection. 

Both the filter samples and composite bloom samples were shipped overnight to SUNY College of Environmental Science and Forestry for analysis. Upon receipt, total chlorophyll and cyanobacterial specific chlorophyll were measured using a bbe Fluoroprobe with a 25 mL cuvette in a workstation format (bbe Moldaenke, Schwentinental, Germany) and 100 mL of bloom material was immediately lyophilized to dryness for the analysis of total toxins. The bbe FluoroProbe divides total chlorophyll into four specific classes based on what accessory pigment excites the chlorophyll fluorescence [125,126,127]. Here, we used phycocyanin-dependent chlorophyll as a measure of cyanobacteria-specific chlorophyll. We did not include phycoerythrin-dependent chlorophyll (the bbe crytophyte channel) in our cyanobacteria-specific chlorophyll since its contribution was minor. Major cyanobacteria species were qualitatively identified in a 500 µL aliquot using an inverted microscope at 50–200×. Identification was not quantitative but instead focused on the presence of certain genera and visual classification of density into subjective categories: very dense, dense, moderate, sparse, and minimal.

### 4.2. Toxin Extraction and Analysis

MCs, ATXs, and PSTs were extracted from filter samples and lyophilized material using 10 mL of 50% methanol containing 1% acetic acid (v/v) and sonicated (3 × 20 s at 32 W). The resulting slurry was centrifuged at 15,000× *g* for 10 min, passed through a 0.45 µm nylon syringe filter, and kept at −20 °C until analysis.

Full methodological details for the analysis of MCs using LC-MS are described in Boyer [128] and Matson et al. [129]. Briefly, solid standards of MC-RR, MC-LR, and MC-LF were purchased from Enzo Life Sciences (Enzo Life Sciences, Farmingdale, NY, USA), calibrated using spectroscopy at 239 nm, and analyzed at the start and end of every batch to ensure microcystin retention times did not drift. As limited reference standards exist for the MCs, the mass spectrometer was tuned to provide an equivalent detector response for these three standards. The MCs: RR, dRR, mRR, H_4_YR, YR, LR, demthyl-LR, AR, FR, WR, LA, LY, LW, LF, and WR were quantified in LR equivalents under the assumption that the response of each compound was equivalent to that of MC-LR. The MC-LR instrument LOD was 4 ng, while the method LODs ranged between 0.10–1 µg/L depending on the volume of sample collected; the response was linear between 1.2 and 250 ng on column, which corresponded to 7.2–2065 µg MC-LR/L in a lake based on a 100 mL sample (slope = 6 × 10^6^, y-int = 4.0 × 10^6^, *R*^2^ = 0.99).

Anatoxin-a was analyzed via LC–MS/MS using a modified version of EPA method 545 that included one quantification and two confirmation ions [27], while homo-anatoxin and dihydro-anatoxin were also analyzed in the same analysis using one quantification and two confirmation ions for each compound (Table S4). Anatoxin-a was purchased from BioMol (Biomol GmbH, Hamburg, Germany), homo-anatoxin-a was purchased from Abraxis (Abraxis LLC, Warminster, PA, USA), and dihydro-anatoxin was obtained from BioMol and also prepared via a sodium borohydride reduction of anatoxin-a, as described in Yang [77]. The structures of the three compounds were confirmed using NMR spectrometry prior to use. Anatoxin-a standards were calibrated gravimetrically. Dihydro-anatoxin and homo-anatoxin were in limited supply; therefore, the quantification of these compounds was performed using response factors relative to the response of anatoxin-a using the transitions in Table S4 [130]. Homo-anatoxin and dihydro-anatoxin were calibrated in MS1 mode scanning molecular weights between 100–450 Da, using extracted ions at 180.1 and 168.2 Da for homo-anatoxin and dihydro-anatoxin, respectively, with anatoxin-a being the calibrant. This calibration assumed that the ionization of the derivatives was similar to that of anatoxin-a. The response factors for the homo-anatoxin and dihydro-anatoxin were determined for the 180.0 → 163.1 Da and 168.0 → 43.1 Da transitions for the two anatoxin derivatives, with relative response factors calculated relative to anatoxin-a. The relative response factors were determined to be 2.74, 4.14, and 0.55 for the quantification of α-dihydro-anatoxin, β-dihydro-anatoxin, and homo-anatoxin relative to anatoxin-a using LC–MS/MS. Dihydro-anatoxin concentrations were reported as a sum of the α and β congeners. Quantification was performed using a linear regression of anatoxin-a with an anatoxin-a standard every 15 samples to verify the stability of the response. Instrument LODs for anatoxin-a, homo-anatoxin, α-dihydro-anatoxin, and β-dihydro-anatoxin were 2, 4, 0.6, and 0.2 pg on column. The corresponding method LODs ranged between 4–10 ng/L, 7–18 ng/L, 1–3 ng/L, and 1–2 ng/L, respectively. The anatoxin-a response was linear between 5 ng and 48 ng on column, which corresponded to 0.96–73.0 µg anatoxin-a/L in a lake based on a 100 mL sample (slope = 8.6 × 10^4^, y-int = 4.0 × 10^4^, R^2^ = 1.0). Dihydro-anatoxin was analyzed for this study between 2015–2017, and homo-anatoxin was measured in 2017. Both anatoxin derivatives were also evaluated in 2018.

PSTs were analyzed using the AOAC 2011.02 post-column chemical oxidation modified for water samples and algal powders [57,131]. The separation was done using a Waters Alliance 2695 solvent delivery system (Waters, Milford, MA, USA) and a Chromenta KB 3µ 150 × 4.6 mm column with an ACE 3µ guard cartridge assembly at 0.8 mL/min (ACE Ltd., Aberdeen, Scotland, UK). The solvent system was: (A) 2 mM heptanesulfonate (Regis Technologies Inc., Morton Grove, IL, USA) in 10 mM ammonium phosphate adjusted to pH 7.1, and (B) 500 mL 2 mM heptane sulfonate in 30 mM ammonium phosphate adjusted to pH 7.1 plus an additional 150 mL of acetonitrile [98]. The separation gradient was: 0% B for 0–3 min, 40% B for 3–5 min, 100% B for 5–13 min, and 100% B for 20 min, followed by equilibration of the column back to 0% B for 10.5 min. Post-column oxidation of the PST ring used 9 mM periodic acid (Alfa Aesar, Ward Hill, MA, USA) in 50 mM potassium phosphate at pH 9 at a flow rate of 0.45 mL/min entering a 25 m 0.25 mm i.d. reaction coil (1 mL total volume) maintained at 65 °C. Following the coil, 0.5 M acetic acid was added at a flow of 0.45 mL/min. PSTs were detected at 330 and 390 nm excitation and emission. PSTs were differentiated from interfering fluorescent compounds by re-injection of the sample with water in place of the oxidant.

To quantitate the PST toxins, primary PST standards were obtained from NRC Canada (Institute for Marine Biosciences, Halifax, NS, Canada) and United States Food and Drug Administration (FDA) (Silver Spring, MD, USA). FDA STX was diluted 1:50 to a concentration of 4 µM prior to use. NRC standards of STX, decarbamoylsaxitoxin, gonyautoxin 1, gonyautoxin 2, gonyautoxin 3, gonyautoxin 4, gonyautoxin 5, decarbamoylgonyautoxin 2, decarbamoylgonyautoxin 3, lyngbyatoxin 1, and C1+C2 were used to calculate the relative response factors. An STX standard was injected every 10 samples to ensure the stability of the response, with PSTs quantified using a linear regression. The STX instrument LODs were 0.9 pg, while the method LODs ranged between 1–2 µg STX/L. The STX response was linear between 9.9 to 198 ng on column, which corresponded to 9.3 and 729.5 µg/L in lake water based on a 100 mL sample (slope = 4.23 × 10^4^; y-int = −5.8 × 10^4^; R^2^ = 0.99). To differentiate PSTs from matrix interferents and naturally fluorescent compounds, samples that contained a peak in the chromatogram were reanalyzed with water in place of the periodic acid oxidant to determine compounds that were fluorescent only after the oxidation of the PST ring system.

Standards of BMAA and diamino butyrate (DAB) were purchased from Sigma Aldrich (Sigma Aldrich, St. Louis, MO, USA), while aminoethyl glycine (AEG) was purchased from Tokyo Chemical Industry (Tokyo, Japan). Free BMAA was measured via LC–MS/MS [129] using a method that cleanly resolves BMAA from the isomeric compounds DAB and AEG that was modified from Lage et al. [132]. The individual sample method detection limits ranged between 1.0–2.6 µg/L. Purified cylindrospermopsin was acquired from the US Environmental Protection Agency (U.S. EPA, Washington D.C., USA). Standards of deoxycylindrospermopsin were purified in-house. Cylindrospermopsins, including cylindrospermopsin, epi-cylindrospermopsin, and deoxy-cylindrospermopsin, were analyzed via LC–MS/MS using a modified version of EPA method 545 [27], as described in Smith et al. [39]. Cylindrospermopsin instrument LODs were 50 pg on column, while methodological LODs ranged between 0.05–0.1 µg/L. The cylindrospermopsin response was linear between 3 to 54 ng on column, which corresponded to 29.8 and 657.9 µg/L in lake water based on a 100 mL sample (slope = 1.16 × 10^5^; y-int = −6.03 × 10^4^; R^2^ = 0.99).

### 4.3. Confirmation of PSTs Using the Saxitoxin Receptor Binding Assay and STX ELISA

Toxin extracts were analyzed for STX-like activity using a receptor binding assay for PSTs by utilizing microplate format methods detailed in Van Dolah et al. [55]. The total toxic potency of a sample was estimated by measuring the competition between radiolabeled STX [11-^3^H] (American Radiolabeled Chemicals, Inc., Saint Louis, MO, USA) and any STX-like activity present in the samples for binding to voltage-gated sodium channels in a crude rat brain membrane preparation. Total PSTs were quantified in terms of STX eq. using a calibration curve prepared from an STX dihydrochloride reference standard acquired from NIST (NIST reference material 8642, National Institutes of Standards and Technology, Gaithersburg, MD, USA). Sample extracts in acidified 50% methanol were analyzed using dilutions of 1/5 (10% methanol) and 1/50 (1% methanol). The method detection limit was 2 µg STX eq./L. 

STX concentrations were determined using Abraxis ELISA (part number 52255B, Abraxis LLC, Warminster, PA, USA) according to the manufacturer’s instructions. Bloom extracts were diluted to a maximum concentration of 5% methanol prior to analysis. Further dilutions were made when the sample concentrations fell outside the linear range of the STX ELISA calibration curve. The STX ELISA method LOD was 0.015 µg STX eq./L.

### 4.4. Calculation of the Photosynthetically Active Radiation, Wind Speed, and Wind Direction

The direct normal irradiance, diffuse horizontal irradiance, wind speed, wind direction, dew point temperature, and solar zenith angle were acquired from the National Renewable Energy Laboratory (NREL) at 42°08′39.5″N, 79°23′07.9″W [133]. PAR was calculated from NREL irradiance values using MODEL-1, developed by Alados et al. [134], for each 30 min point, and averaged over two-week intervals starting from 1 January 2014 until 31 December 2017. Wind speed and wind angle data consisting of wind speed and wind direction at 30 min intervals were projected from cardinal coordinates into Cartesian coordinates, with each point converted into east/west or north/south proportions of wind speed. The AWD and AWS for each two-week interval starting from 1 January 2014 were determined from the average east/west and north/south wind speeds. Daily rainfall was acquired from the Chautauqua Lake-Jamestown airport Meteorological Aerodrome Report (METAR) weather station (42°07′48.0″N, 79°13′48.0″W) and averaged over two-week intervals starting 1 January 2014.

### 4.5. Model Simplification and Selection Approach for Blue-Green Algal Chlorophyll and Cyanotoxins

Models for cyanobacteria toxins and cyanobacteria abundance were developed based on the methods described by Bunea et al. and Abram et al. [135,136]. The statistical methods used a combination of bootstrap enhanced LASSO and ridge regression, as described in the supplemental Technical Note. These regression techniques use a penalization term that estimates the likelihood of any particular variable’s inclusion into a model through bootstrap iterations. This is done by separating the data into equal-sized subsets that are used to create a training set versus a separate test set that can be used to evaluate the model. The importance of each predictor is evaluated by comparing the most complex model, which includes all predictors, to the most simple model containing only an intercept and no predictors.

Penalized regression models were used here because, following the removal of any data interval that had missing values for any parameter, only a limited number of points were left for generating the model (Table S5). Due to over-parameterization, penalized regression models were used in place of standard multiple regression techniques. While the low sample size did not impact some parameters through over-parameterization (cyanobacterial chlorophyll) and was only a small constraint for others (MCs), it resulted in full models becoming over-parameterized for some response variables (PSTs and ATXs). Because standard regression techniques could not be used with response variables that had become heavily over-parameterized, our approach allowed us to systematically produce models for each response variable. A more detailed summary of the penalized regression approach is described in the supplemental Technical Note.

## Figures and Tables

**Table 1:** Water quality measurements collected in the North and South Basins of Chautauqua Lake at biweekly intervals over 2014–2017. Statistical differences between water quality parameters were determined with paired *t*-tests. Significant differences (*p* < 0.05) are in bold. Water quality parameters are given as an average of 32 samples collected at each site and error shows one standard deviation from the mean. Chlorophyll differences were evaluated following natural log transformation due to extreme violations of normality (see Section 2.1 for further discussion). TN—total nitrogen, TP—total phosphorus.

Basin	North Basin (Mean ± SD)	South Basin (Mean ± SD)	Paired *t*-test Difference in Means (*p*-Value)
TP (μg P/L)	41.7 ± 18.4	68.9 ± 35.1	**27.1 (<0.0001)**
TN (mg N/L)	0.57 ± 0.68	0.74 ± 0.33	0.166 (0.15)
Ammonia (mg N/L)	0.046 ± 0.057	0.034 ± 0.032	0.012 (0.29)
NO_2_^−^ + NO_3_^−^ (mg N/L)	0.029 ± 0.028	0.026 ± 0.038	0.004 (0.66)
Water Temp (°C)	22.4 ± 2.3	23.5 ± 2.6	**0.66 (0.039)**
pH	7.93 ± 0.39	8.07 ± 0.66	0.13 (0.23)
Secchi disk depth (m)	2.29 ± 0.95	1.09 ± 0.70	**1.17 (<0.0001)**
Conductivity (μS)	190.4 ± 33.3	193 ± 31.7	3.35 (0.57)
Total chlorophyll (µg/L)	2426 ± 16,780	700 ± 3370	NA *
Cyanobacterial chlorophyll (µg/L)	2408 ± 16,780	635 ± 3080	NA *
ln(Total chlorophyll + 1) *	3.1 ± 2.3	3.8 ± 2.0	0.70 (<0.001) *
ln(Cyanobacterial chlorophyll + 1) *	2.5 ± 2.6	3.3 ± 2.3	0.78 (<0.001) *

* Chlorophyll differences were evaluated using a two-sample Welch test on the natural-log-transformed total and cyanobacterial chlorophyll concentrations due to the deviations from normality.

**Table 2:** Chlorophyll fluoroprobe measurements for cyanobacterial specific chlorophyll for the highest quartile of blooms in Chautauqua Lake. Error shows one standard deviation from the mean.

Basin	Number of Samples	Range (µg/L)	Mean ± SD (µg/L)	Median (µg/L)
North	86	32.4–210,100	9590 ± 32,610	212
South	49	97.4–29,700	2490 ± 5830	373

**Table 3:** The occurrence and concentrations of microcystins (MCs), anatoxins, and paralytic shellfish poisoning toxins (PSTs) in Chautauqua Lake as evaluated by year. Units for the toxin concentrations are reported as total MCs (µg/L), total anatoxins (ATXs) (µg/L), and total PSTs (µg STX eq./L). Only anatoxin-a concentrations are shown since dihydro-anatoxin was only detected in one sample and homo-anatoxin was not detected in Chautauqua Lake.

Year	Number of Samples Collected	Date Range	Microcystins				Anatoxins				Paralytic Shellfish Poisoning Toxins			
			Median	Mean	SD	Number of Toxic Samples	Median	Mean	SD	Number of Toxic Samples	Median	Mean	SD	Number of Toxic Samples
2014	137	22 June–13 October	2.8	17	25	11	0.88	1.2	1.2	12	NA	NA	NA	NA
2015	128	07 June–19 October	5.1	101	325	23	0.058	0.34	0.46	23	NA	NA	NA	NA
2016	144	29 May–17 October	4.8	54	211	28	0.36	0.91	1.5	53	8.3	17	25	14
2017	211	29 May–28 November	3.1	192	841	95	0.033	0.052	0.075	7	24	46	98	32

**Table 4:** A summary of some of the models produced for cyanobacterial chlorophyll (Chl), microcystins, anatoxin-a, and paralytic shellfish poisoning toxins. Full model outputs are shown in Figures S4–S7, while an explanation of the parameter selection is described in the text and the supplemental Technical Note. Abbreviations: AWD—average wind direction, AWS—average wind speed, PAR—photosynthetically active radiation

Location	Model Predictors			
	Cyanobacterial Chlorophyll (Chl)	Microcystins	Anatoxins	Paralytic Shellfish Poisoning Toxins
Lakewide	pH, TP, TN	None	Chl, PAR, AWS *	Chl, AWS, conductivity
North Basin	pH, TP, Secchi	Chl, TP, water temp, rainfall	-	AWS, rainfall, Secchi
South Basin	None	Chl, PAR, AWD	Chl, AWD, water temp, Secchi	Chl, AWS, rainfall, Secchi

* The majority of anatoxin-a detections were in the South Basin, with the North Basin anatoxin-a largely limited to detections at one site (Children’s Beach, Table S1) in 2017.

## Supplementary Materials

The following are available online at https://www.mdpi.com/2072-6651/12/9/559/s1, Table S1: GPS coordinates for all sites sampled on Chautauqua Lake for cyanobacteria toxins, Table S2: List of algal genera that were detected by visual examination in Chautauqua Lake in the years 2014–2017, Figure S1: Sum of all the microcystins quantified throughout a yearly sampling season broken down by the proportion of the individual microcystin congeners detected, Figure S2: Yearly and temporal concentrations of microcystins, anatoxins, and paralytic shellfish toxins in Chautauqua Lake, New York, Figure S3: Yearly and temporal concentrations of samples containing lower concentrations of microcystins in Chautauqua Lake, New York, Table S3: Summary of the number of samples that contained microcystins (MCs), anatoxin-a, and paralytic shellfish poisoning toxins (PSTs) from the CLA, Bridge, and Whiteside sites between 2014–2017, Figure S4: Penalized regression models for cyanobacterial chlorophyll in the lakewide (**A** and **B**), the North Basin (**C** and **D**), and the South Basin (**E**), Figure S5: Penalized regression models for Microcystins (MCs) in the whole lake (**A**), the North Basin (**B** and **C**) and the South Basin (**D** and **E**), Figure S6: Penalized regression models for paralytic shellfish poisoning toxins (PSTs) (**A** and **B**), and anatoxin-a (**C** and **D**) in both basins, Figure S7: Penalized regression models for paralytic shellfish poisoning toxins (PSTs) in the North Basin (**A** and **B**) and the South Basin (**C** and **D**), and anatoxin-a in the South Basin (**E** and **F**), Table S4: Multiple reaction monitoring (MRM) transitions used for the detection of anatoxin, homo-anatoxin, dihydro-anatoxin, cylindrospermopsin, and deoxycylindrospermopsin, Technical Note: A description of the penalized regression models and a description of the challenges related to traditional statistical analysis of cyanobacterial chlorophyll and toxins in Chautauqua Lake, Table S5: The number of points, representing two-week averages for each parameter, used to produce each model shown in Table 4 and Figures S7–S10 following omission of any data points that contained any missing value.
