# Supplementary material for: Limnological Differences in a Two-Basin Lake Help to Explain the Occurrence of Anatoxin-a, Paralytic Shellfish Poisoning Toxins, and Microcystins
Source: Toxins (Basel). 2020 Aug 30;12(9):559. doi: 10.3390/toxins12090559 (PMC7551069; doi:10.3390/toxins12090559)
Supplement: Supplementary file 1 [file toxins-12-00559-s001.pdf]

Differences in Limnology in a Two-Basin Lake Help Explain the Occurrence of Anatoxin-a, Paralytic Shellfish Poisoning Toxins, and Microcystins

Zacharias J. Smith, Douglas E. Conroe, Kimberly L. Schulz, and Gregory L. Boyer

Table S1. GPS coordinates for all sites sampled on Chautauqua Lake for cyanobacteria toxins, as shown in Figure 1.

Site Name	Site GPS Coordinates Location
Chautauqua CSLAP North	42°10'51.4"N 79°25'50.5"W
Chautauqua CSLAP South	42°07'23.8"N 79°21'50.0"W
Bemus Bay Shore Edge	42°09'38.3"N 79°23'33.9"W
Bemus Hotel Lenhart	42°09'31.7"N 79°23'41.9"W
Bemus Point DEC Launch Ramp	42°09'22.5"N 79°23'36.5"W
Boys & Girls Club Children's Camp - Chautauqua Institution	42°12'14.6"N 79°27'37.1"W
Bridge	42°09'08.4"N 79°23'06.6"W
Celoron Launch/Lighthouse	42°06'40.7"N 79°16'58.3"W
Central Dock	42°12'33.7"N 79°27'44.2"W
Cheney Ave Dock	42°07'18.8"N 79°19'31.4"W
Children's Beach	42°12'39.3"N 79°27'43.6"W
CLA	42°06'09.5"N 79°18'05.8"W
Crosswinds Marina	42°13'31.8"N 79°26'22.4"W
Approximate Location of Drinking Water Intake	42°12'45.9"N 79°27'56.8"W
Heinz Beach	42°12'21.4"N 79°27'38.8"W
Lakewood Beach	42°06'24.4"N 79°19'35.2"W
Long Point Park Beach	42°10'19.7"N 79°24'46.1"W
Long Point Park Marina	42°10'19.8"N 79°24'56.0"W
Mayville Beach	42°14'31.3"N 79°29'39.5"W
Pier Beach	42°12'38.9"N 79°27'44.8"W
Prendergast Point	42°11'21.7"N 79°26'27.0"W
Stow Ferry	42°09'25.7"N 79°24'01.9"W
University Beach	42°12'49.3"N 79°28'09.1"W
Whiteside	42°11'38.5"N 79°25'16.4"W
Woodlawn Dock	42°10'16.5"N 79°25'55.0"W

Table S2. List of algal genera that were detected by visual examination in Chautauqua Lake in the years 2014-2017.

Year	Detected Algal Genera	Detected Algal Genera
2014		
	Asterionella	*Melosira*
	Aulacoseria	*Microcystis sp.*
	Ceratium	*Microseira* (*Lyngbya*)
	Closterium	*Pediastrum*
	Cuspidothrix (*Aphanizomeneon*)	*Stephanodiscus*
	Dinobryon	*Straurastrum*
	Dolichospermum	*Woronichinia*
	Fragilaria	*Gloeotrichia*
2015		
	Asterionella	*Melosira*
	Aulacosiria	*Microcystis sp.*
	Ceratium	*Pediastrum*
	Cuspidothrix (*Aphanizomeneon*)	*Straurastrum*
	Dinobryon	*Volvox*
	Dolichospermum	*Woronichinia*
	Fraglaria	
2016		
	Arthrospira	*Microspora*
	Asterococcus	*Mougeotia*
	Astrionella	*Nostoc*
	Ceratium	*Oedogonium*
	Chrysosphaerella	*Oscillatoria*
	Cuspidothrix (*Aphanizomeneon*)	*Pediastrium*
	Dinobryon	*Planktothrix*
	Dolichospermum	*Spherocystis*
	Fragilaria	*Spirogyra*
	Gloeotrichia	*Straurastrum*
	Limnothrix	*Synedra*
	Meridion	*Tribonema*
	Merisopedia	*Woronichinia*
		Microspora
2017		
	Aphanocapsa	*Microcystis*
	Asterionella	*Mougeotia*
	Ceratium	*Pediastrum*
	Closterium	*Planktothrix*
	Coelastrum	*Scenedesmus*
	Coocmyxa	*Selenastrum*
	Cuspidothrix (*Aphanizomeneon*)	*Sphaeocystis*
	Desmodesmus	*Straurastrum*
	Dinobryon	*Woronichinia*
	Dolichospermum	*Gloeotrichia*

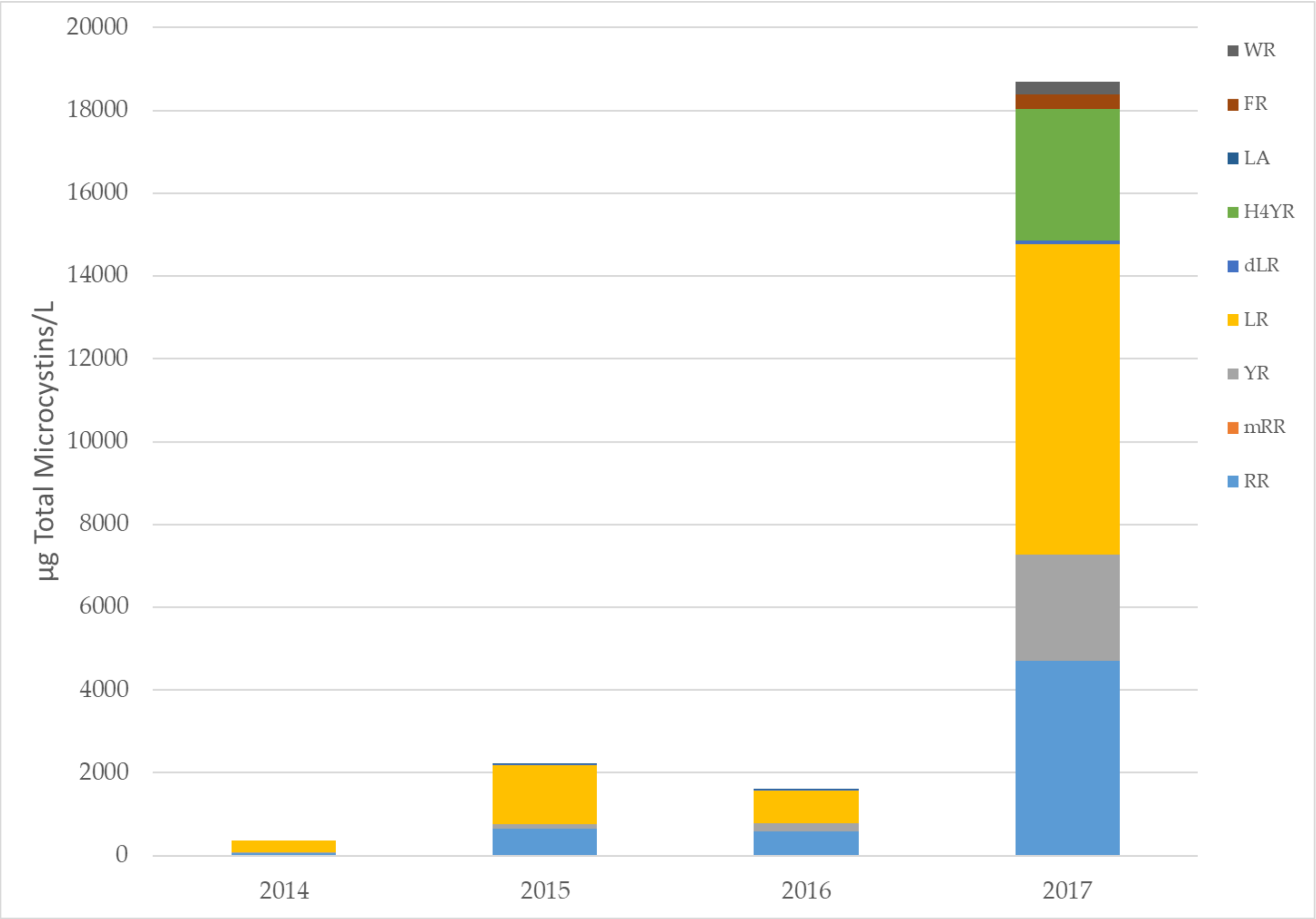

Figure S1. Sum of all the microcystins quantified throughout a yearly sampling season broken down by the proportion of the individual microcystin congeners detected. The most common variants detected were RR, YR, and LR. Congeners not detected are not shown. Several of the congeners were not detected in multiple years.

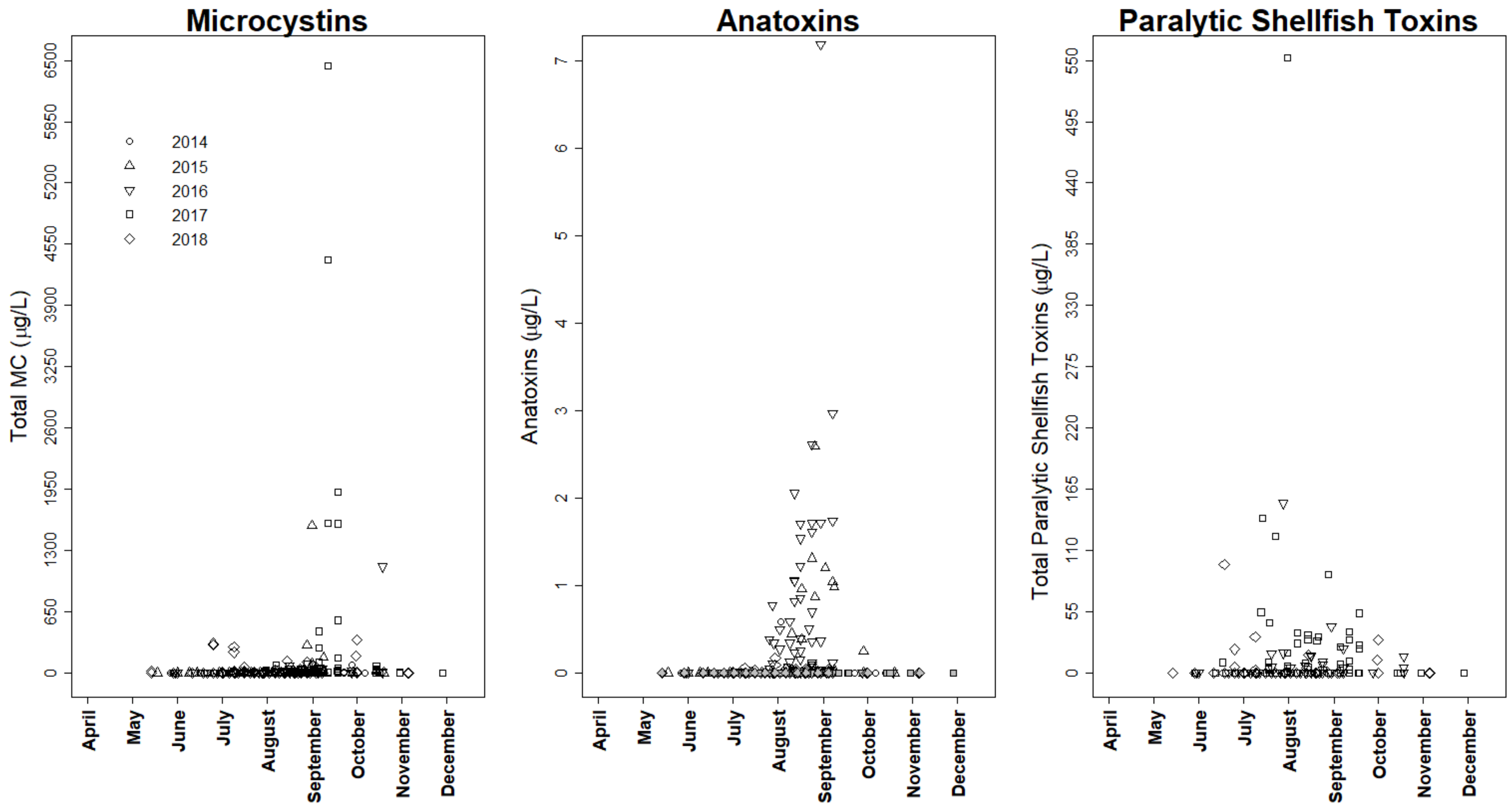

Figure S2. Yearly and temporal concentrations of microcystins, anatoxins, and paralytic shellfish toxins in Chautauqua Lake, New York. Homo-anatoxin and dihydro-anatoxin are shown grey-filled, while anatoxin-a is shown as open symbols.

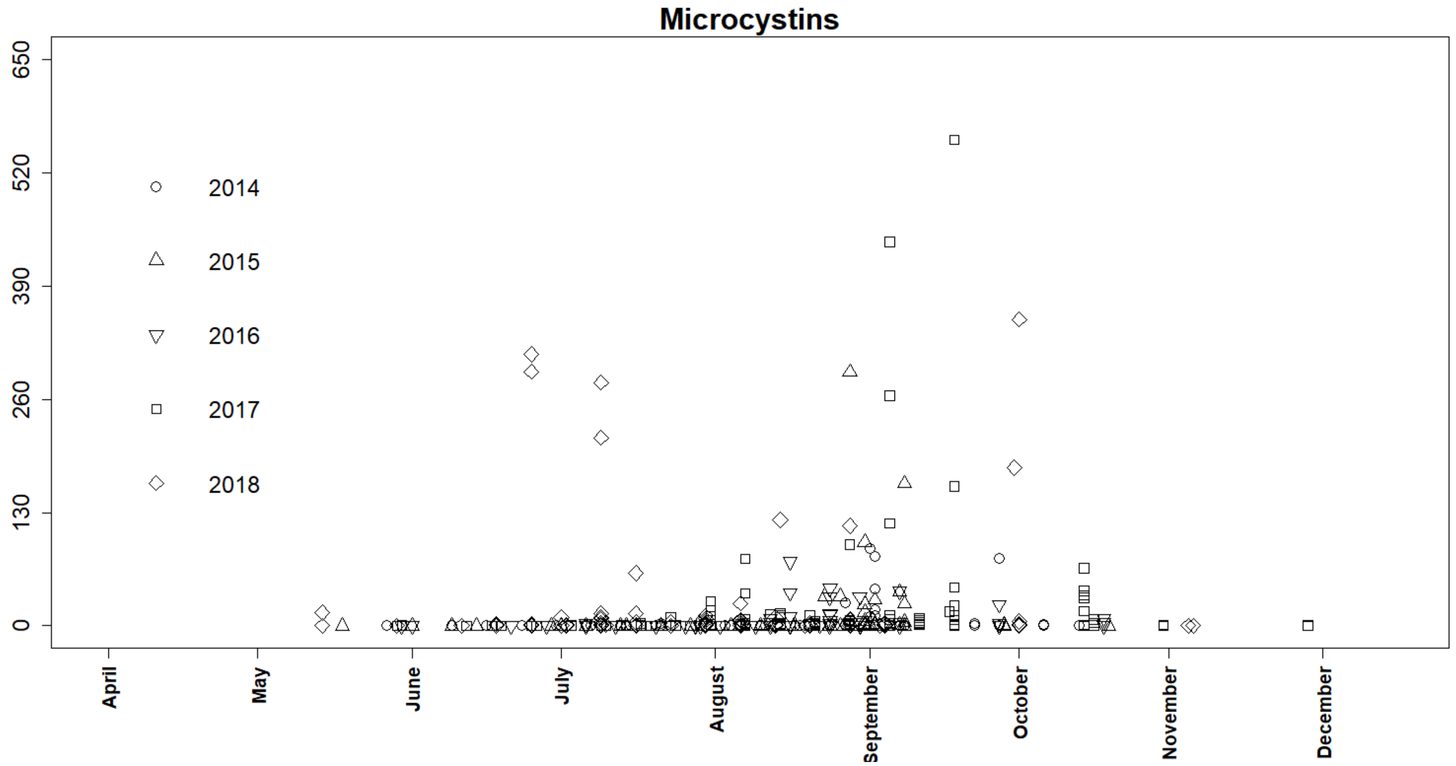

Figure S3. Yearly and temporal concentrations of samples containing lower concentrations of microcystins in Chautauqua Lake, New York. Y-axis has been scaled to better show the heterogeneity of samples.

Table S3. Summary of the number of samples that contained microcystins (MCs), anatoxin-a, and paralytic shellfish poisoning toxins (PSTs) from the CLA, Bridge, and Whiteside sites between 2014-2017. NA, not tested. Num, number.

Year	**CLA**			**Bridge**			**Whiteside**		
	Num. Samples with MCs/total samples	Num. with anatoxin-a	Num. with PSTs	Num. Samples with MCs/total samples	Num. with anatoxin-a	Num. with PSTs	Num. Samples with MCs/total samples	Num. with anatoxin-a	Num. with PSTs
2014	1/12	0	NA	3/13	1	NA	0/17	0	NA
2015	4/18	5	NA	2/14	1	NA	1/14	0	NA
2016	3/17	9	3	4/14	2	3	1/17	0	1
2017	8/17	1	3	13/18	0	4	7/18	0	5

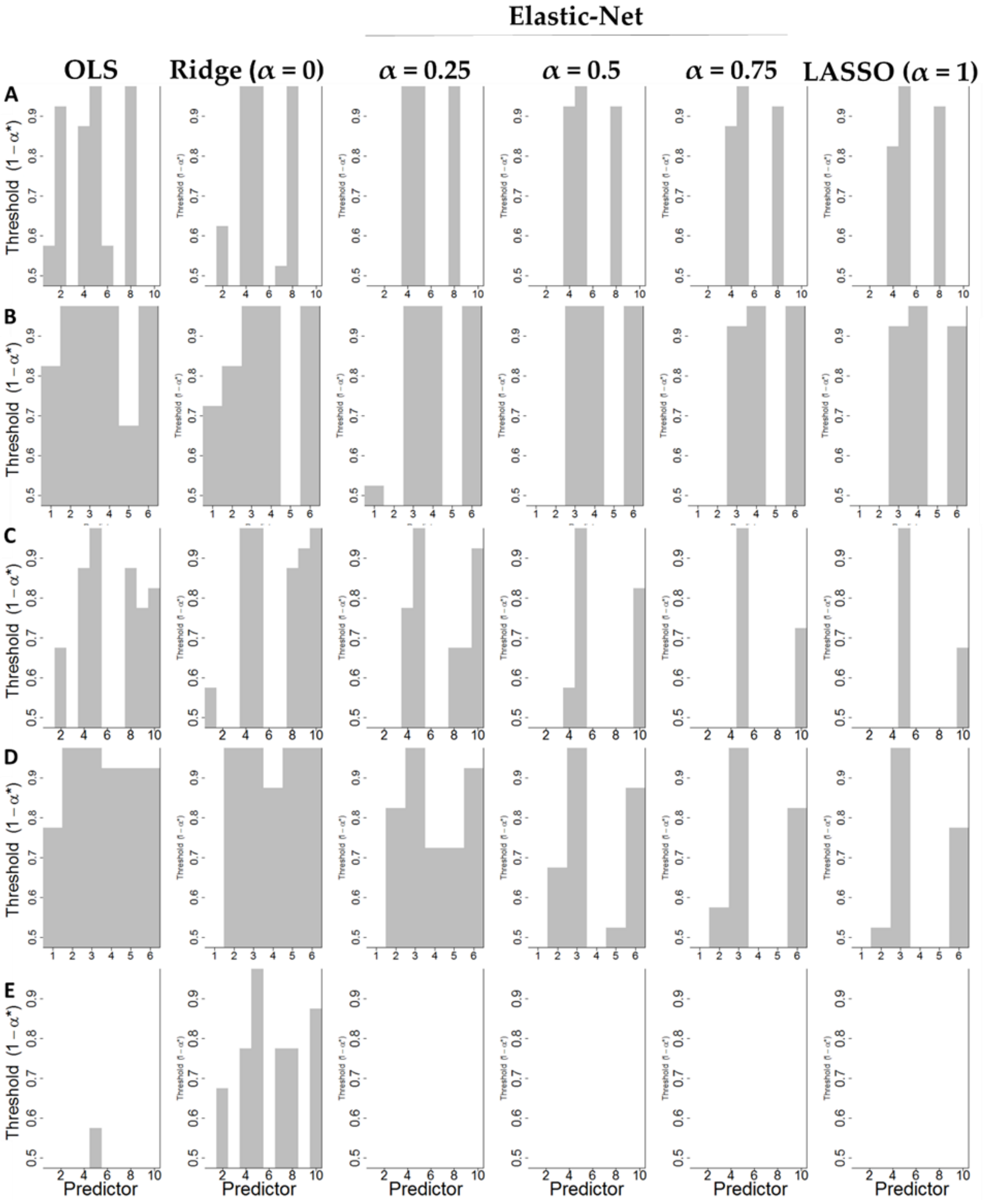

Figure S4. Penalized regression models for cyanobacterial chlorophyll in the lakewide (**A** and **B**), the North Basin (**C** and **D**), and the South Basin (**E**). The models are shown as follows: **A**, Whole lakewide model; **B**, Whole lake with OLS terms selected in **A**; **C**, North Basin full model; **D**, North Basin with OLS terms selected in **C**; **E**, South Basin full model. As only one term was selected for the South Basin full model, no further analysis could be performed. Predictors used for **A**, **C**, **E**: 1 Photosynthetically Active Radiation (PAR), 2 Average Wind Speed (AWS), 3 Average Wind Direction (AWD), 4 pH, 5 Total Phosphorus (TP), 6 Conductivity, 7 Water Temperature, 8 Total Nitrogen (TN), 9 Rainfall, 10 Secchi Disk Depth. Predictors used for **B**: 1 PAR, 2 AWS, 3 pH, 4 TP, 5 Conductivity, 6 TN. Predictors for **D**: 1 AWS, 2 pH, 3 TP, 4 TN, 5 Rainfall, 6 Secchi Disk Depth.

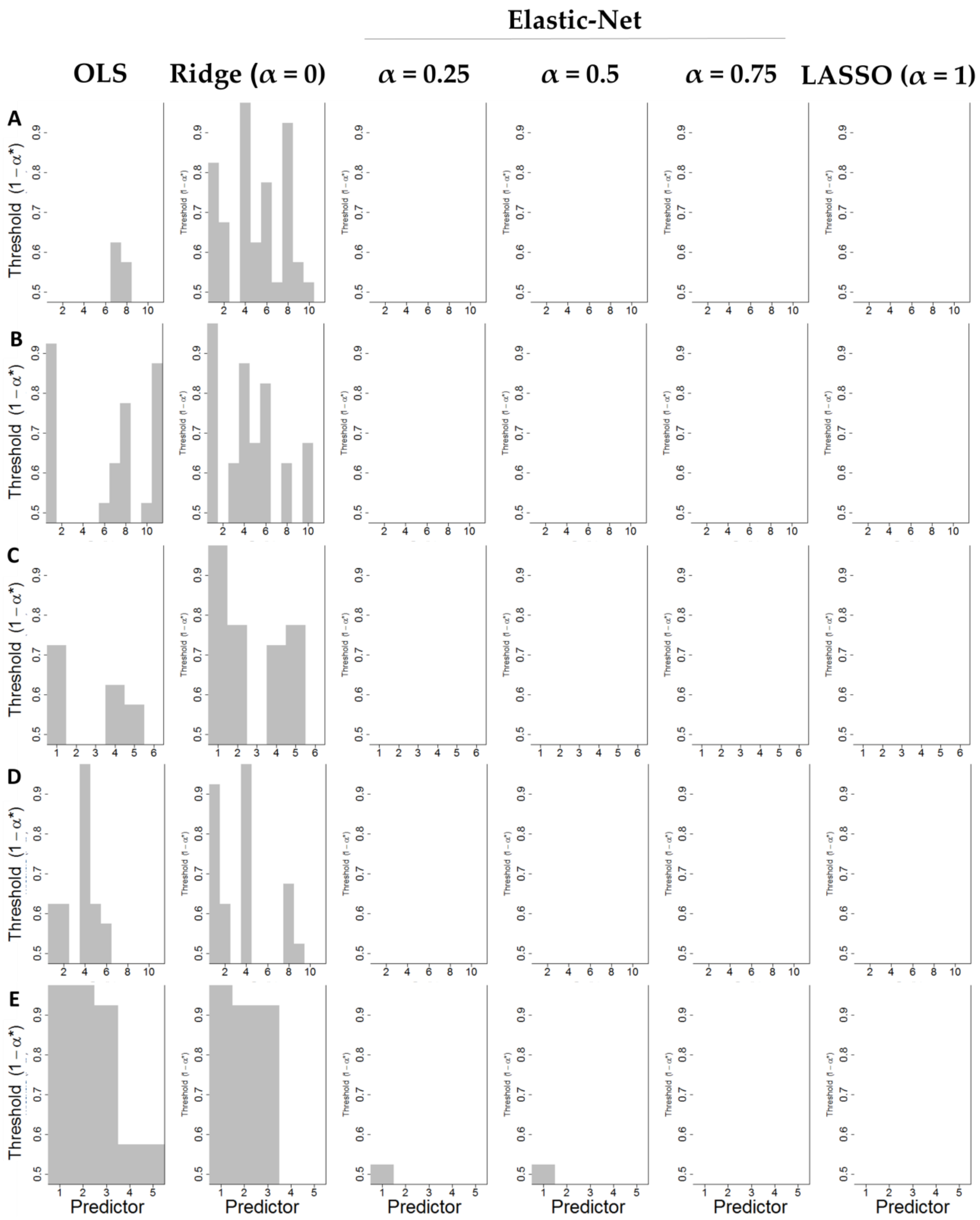

Figure S5. Penalized regression models for Microcystins (MCs) in the whole lake (**A**), the North Basin (**B** and **C**) and the South Basin (**D** and **E**). The models are shown as follows, **A**, whole lake full model; **B**, North Basin full model; **C**, North Basin with OLS terms selected in **B**; **D**, South Basin full model; **E**, South Basin with OLS terms selected in **D**. For brevity, the penalized models for the full lake are not shown as neither of the terms chosen in **A** were selected in the penalized models. Predictors used for **A**, **B**, and **D**: 1 Log Cyanobacterial Chlorophyll, 2 Photosynthetically Active Radiation (PAR), 3 Average Wind Speed (AWS), 4 Average Wind Direction (AWD), 5 pH, 6 Total Phosphorus (TP), 7 Conductivity, 8 Water Temperature, 9 Total Nitrogen (TN), 10 Rainfall, 11 Secchi Disk Depth. Predictors used for **C**: 1 Log Cyanobacterial Chlorophyll, 2 TP, 3 Conductivity, 4 Water Temperature, 5 Rainfall, 6 Secchi Disk Depth. Predictors used for **E**: 1 Log Cyanobacterial Chlorophyll, 2 PAR, 3 AWS, 4 Water Temperature, 5 TN, 6 Rainfall.

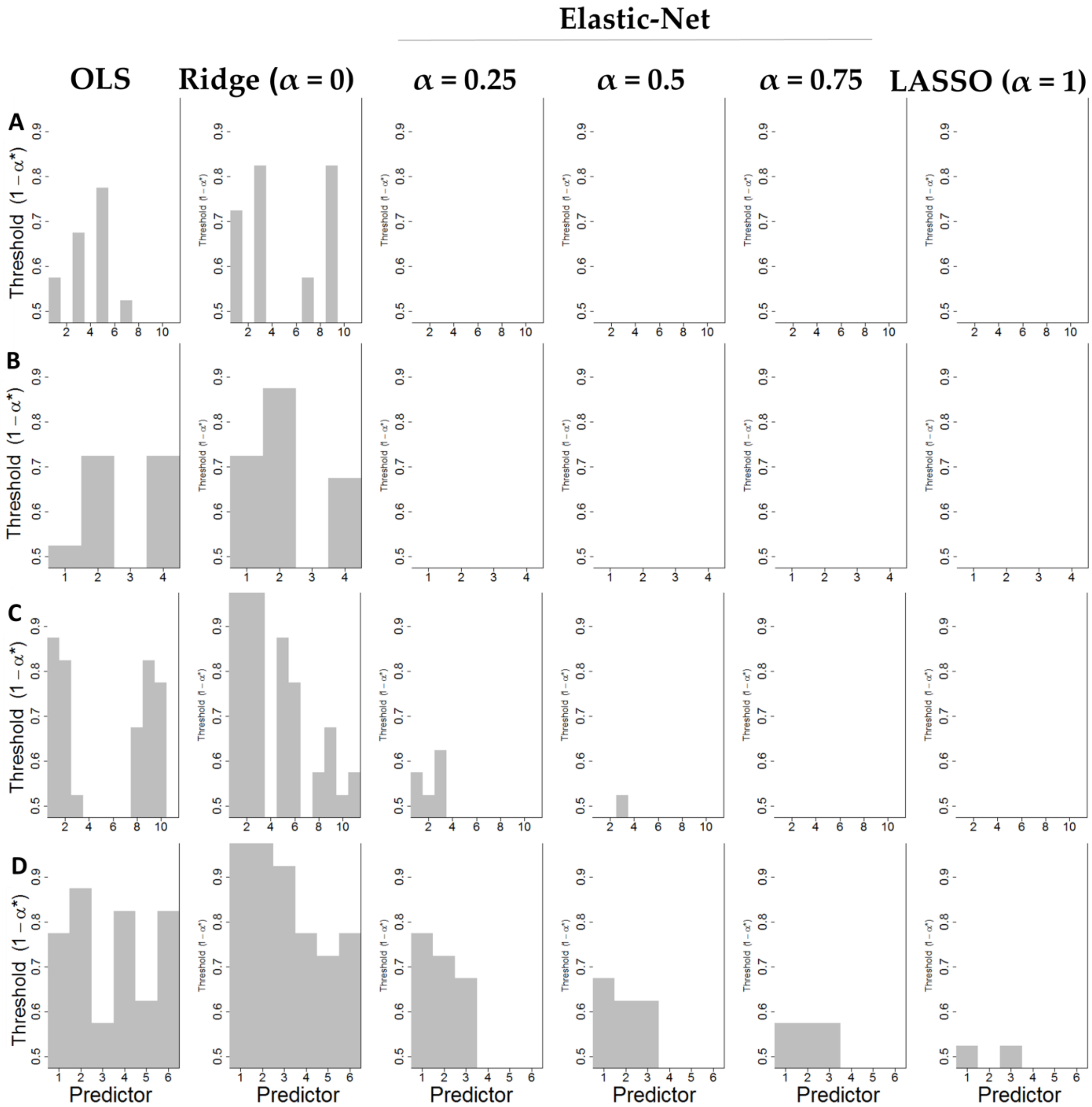

Figure S6. Penalized regression models for paralytic shellfish poisoning toxins (PSTs) (**A** and **B**), and anatoxin-a (**C** and **D**) in both basins. The models are shown as follows, **A**, PSTs full model; **B**, PSTs with OLS terms selected in **A**; **C**, anatoxin-a full model; **D**, anatoxin-a terms selected in **C**. Predictors used for **A** and **C**: 1 Log Cyanobacterial Chlorophyll, 2 Photosynthetically Active Radiation (PAR), 3 Average Wind Speed (AWS), 4 Average Wind Direction (AWD), 5 pH, 6 Total Phosphorus (TP), 7 Conductivity**,** 8 Water Temperature**,** 9 Total Nitrogen (TN), 10 Rainfall, 11 Secchi Disk Depth. Predictors used for **B**: 1 Log Cyanobacterial Chlorophyll, 2 AWS, 3 pH, 4 Conductivity. Predictors used for **D**: 1 Log Cyanobacterial Chlorophyll, 2 PAR, 3 AWS, 4 Water Temperature, 5 TN, 6 Rainfall.

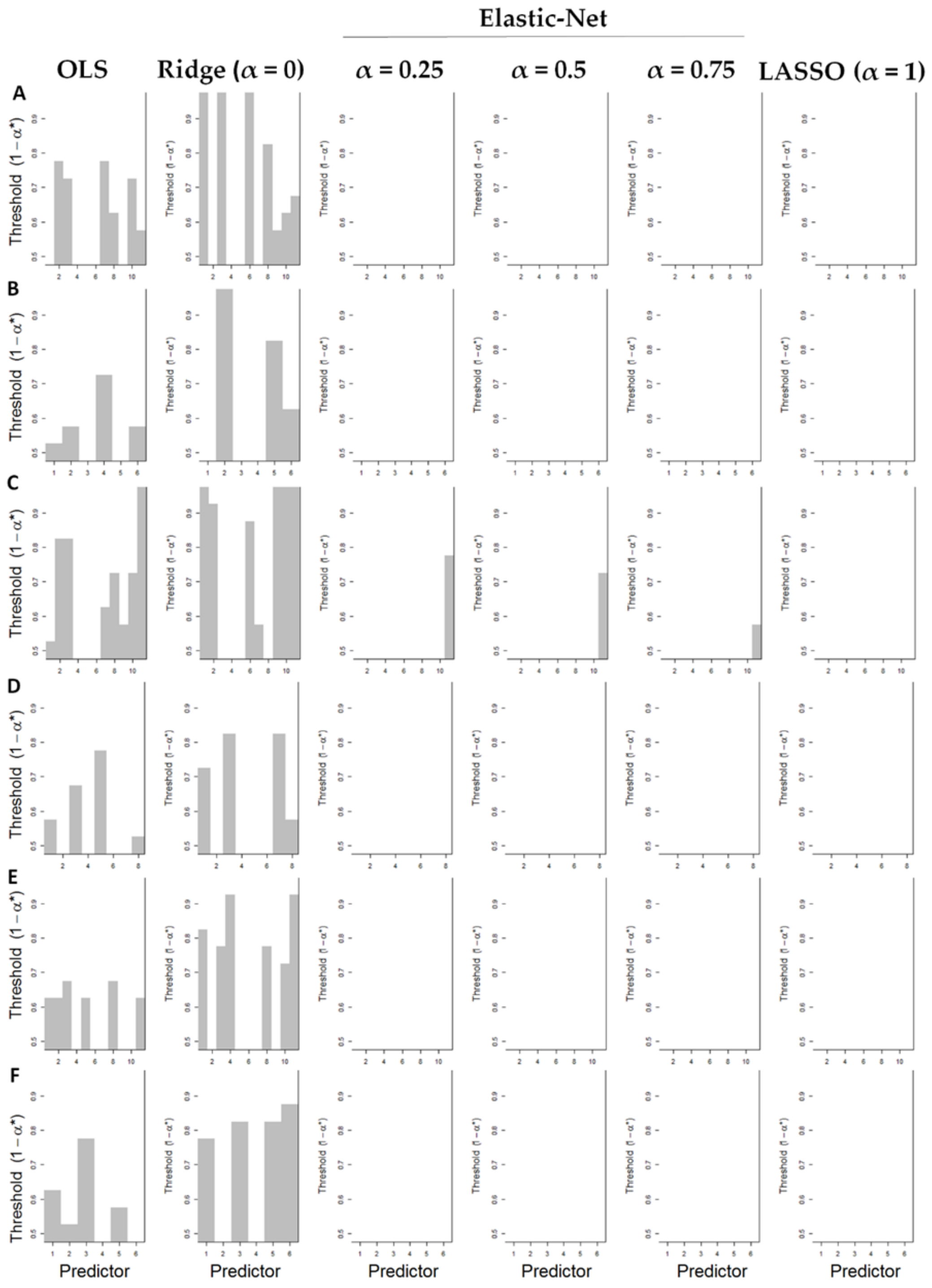

Figure S7. Penalized regression models for paralytic shellfish poisoning toxins (PSTs) in the North Basin (**A** and **B**) and the South Basin (**C** and **D**), and anatoxin-a in the South Basin (**E** and **F**). The models are shown as follows, **A**, PSTs North Basin full model; **B**, PSTs North Basin with OLS terms selected in **A**; **C**, PSTs South Basin full model; **D**, PSTs South Basin with OLS terms selected in **D**; **E**, anatoxin-a South Basin full model; **F**, anatoxin-a South Basin with OLS terms selected in **E**. Predictors used for **A**, **C**, and **E**: 1 Log Cyanobacterial Chlorophyll, 2 Photosynthetically Active Radiation (PAR), 3 Average Wind Speed (AWS), 4 Average Wind Direction (AWD), 5 pH, 6 Total Phosphorus (TP), 7 Conductivity, 8 Water Temperature, 9 Total Nitrogen

(TN), 10 Rainfall, 11 Secchi Disk Depth. Predictors used for **B**: 1 PAR, 2 AWS, 3 Conductivity, 4 Water Temperature, 5 Rainfall, 6 Secchi Disk Depth. Predictors used for **D**: 1 Log Cyanobacterial Chlorophyll, 2 PAR, 3 AWS, 4 Conductivity, 5 Water Temperature, 6 TN, 7 Rainfall, 8 Secchi Disk Depth. Predictors used for **F**: 1 Log Cyanobacterial Chlorophyll, 2 PAR, 3 AWS, 4 pH, 5 Water Temperature, 6 Secchi Disk Depth.

Table S4. Multiple reaction monitoring (MRM) transitions used for the detection of anatoxin, homo-anatoxin, dihydro-anatoxin, cylindrospermopsin, and deoxycylindrospermopsin.

Toxin	Quantitation Transition	Confirmation Transitions
Anatoxin-a	166.0 → 131.0	166.0 → 148.0; 166.0 → 90.90
Homo-anatoxin	180.0 → 163.1	180.0 → 145.1; 180.0 → 105.0
Dihydro-anatoxin	168.0 → 43.1	168.0 → 55.9, 168.0 → 67.0
Cylindrospermopsin	416.1 → 194.0	416.1 → 274.4, 416.1 → 336.2
Deoxycylindrospermopsin	400.1 → 194.3	400.1 → 274.5, 400.1 → 320.5

Technical Note A description of the penalized regression models and a description of the challenges related to traditional statistical analysis of cyanobacterial chlorophyll and toxins in Chautauqua Lake.

The penalized regression modeling approach was chosen over more traditional methods as it resolved several common issues with regression that were observed in the Chautauqua Lake dataset. In the multiple regression MC models that contained many predictors, there were small but detectable amounts of multi-collinearity, as identified by variable inflation factor (VIF) and condition indices. Although simplified models did not appear to contain multicollinearity, standard model simplification approaches, as a side effect of their reliance on *p*-values, will be influenced by the instability of coefficients when collinear terms are included in the same model. For these reasons we determined that standard model simplification using stepwise removal or addition of predictors and F-tests or AIC for model selection would not be reliable when trying to answer the questions we wanted to explore in Chautauqua Lake.

To compare the results from the four response variables, the model selection approach for each response, cyanobacterial chlorophyll, MCs, ATX, and PSTs started with the same predictors. Initial model simplification was performed using bootstrap enhanced OLS regression using 11 predictors for the toxins and 10 for cyanobacterial chlorophyll, with any of the parameters selected in 50% or more of the OLS models by non-parametric bootstrap quantile confidence interval selection (QNT) [110] included in a further set of penalized regression models. This initial simplification was performed because the inclusion of extraneous terms in the full models impacted the inclusion probability for each of the other parameters. Because the full models only selected a limited number of predictors, a reduction in the number of included terms to only those selected in the full model iteration of OLS led to better convergence at higher model restrictiveness levels in the penalized regression models.

Using the parameters selected by the bootstrapped OLS model, five penalized regression models ridge (α= 0), elastic net (α= 0.25, 0.5, 0.75) and LASSO (α = 1) were produced at different levels of selectivity (α), with larger α increasing the selectivity criteria for inclusion of parameters into each model. Parameter inclusion for each model was evaluated using QNT selection [110]. Because ideal model restrictiveness levels varied between response variables, each model from α = 0 to α = 1 is shown in Figures S6.5, S6.6 and S6.7. QNT parameter selection allowed for a meaningful interpretation of ridge regression, where unimportant parameters were eliminated due to their lack of contribution to the model. Rather than only reducing the impact of unimportant coefficients on ridge regression models, QNT selection produced ridge models with limited numbers of predictors. All models were used to examine the differences between the basins and between toxins only, not to produce models capable of prediction.

Table S5. The number of points, representing two-week averages for each parameter, used to produce each model shown in Table 4 and Figures S7–S10 following omission of any data points that contained any missing value. The maximum number of possible data points was 40. Chl, cyanobacterial chlorophyll.

Toxin	Number of Input Points for Model
Chl All Lake	31
Chl All Lake OLS only	31
Chl North	30
Chl North OLS only	30
Chl South	23
Chl South OLS only	NA
MC All Lake	19
MC All Lake OLS only	19
MC North	12
MC North OLS only	13
MC South	14
MC South OLS only	16
PSTs	12
PSTs OLS only	12
PSTs North	10
PSTs North OLS only	10
PSTs South	10
PSTs South OLS only	10
ATX	12
ATX OLS Only	12
ATX South	14
ATX South OLS Only	14
